# Is low-level laser therapy effective for patients with knee joint osteoarthritis? implications and strategies to promote laser therapy usage

**DOI:** 10.3389/fbioe.2022.1089035

**Published:** 2022-12-08

**Authors:** Mohammad Adib Khumaidi, Idrus Paturusi, Nury Nusdwinuringtyas, Andi Asadul Islam, William Ben Gunawan, Fahrul Nurkolis, Nurpudji Astuti Taslim

**Affiliations:** ^1^ Orthopedic and Traumatology, Hasanuddin University School of Medicine, Makassar, Indonesia; ^2^ Faculty of Medicine and Health, Universitas Muhammadiyah Jakarta, Jakarta, Indonesia; ^3^ Physical Medicine and Rehabilitation Department, Faculty of Medicine Universitas Indonesia/Cipto Mangunkusumo Central Hospital, Jakarta, Indonesia; ^4^ Nutrition Science Department, Faculty of Medicine, Diponegoro University, Semarang, Indonesia; ^5^ Biological Sciences, State Islamic University of Sunan Kalijaga Yogyakarta, Yogyakarta, Indonesia; ^6^ Clinical Nutrition, Faculty of Medicine, Hasanuddin University, Makassar, Indonesia

**Keywords:** low-level laser therapy, osteoarthritis, knee joint, cold laser therapy, photobiomodulation, light-emitting diodes, exercise, molecular biophysics

## 1 Introduction

Osteoarthritis (OA) is a chronic, degenerative joint disease marked by clinical symptoms and joint tissue deformation that predominantly harms joint cartilage, resulting in discomfort, edema, and stiffness near the joint (Jang, Lee, and Ju, 2021). The knee was known as the biggest synovial joint in humans which is made up of synovium, infrapatellar fat pad, ligaments, and bone components and experiences a lot of use and stress; making it a common location for painful disorders, notably OA (knee osteoarthritis; KOA) (Mora, Przkora, and Cruz-Almeida, 2018). KOA is a degenerative, inflammatory condition that affects knee joint and is accompanied by discomfort, impairment, and a lower quality of life (Vitaloni et al., 2019). Increased inflammatory activity has been associated with pain, eventhough the relationship level vary (Dainese et al., 2021). Almost 85% of OA incidences were correlated with KOA (Vos, T. et al., 2016). The prevalence of KOA among asymptomatic uninjured knees were ranging from 4–14% in less than 40 years age adults and 19–43% in adults aged 40 years and over (Culvenor et al., 2019).

Some conservative intervention options for KOA are exercise therapy, non-steroidal anti-inflammatory drugs (NSAIDs), and low-level anti-inflammatory laser therapy (LLLT). In comparison to standard care after 8 weeks, exercise dramatically reduces pain and enhances function, performance, and quality of life in persons with KOA (Goh et al., 2019) However, exercise therapy may be more beneficial for patients who are younger and are not in the waiting period for joint replacement. NSAIDs are recommended in most clinical treatment guidelines KOA, although the intake of these drugs is associated with adverse effects (Rannou, Pelletier, and Martel-Pelletier, 2016). Although some NSAID doses have been demonstrated to be beneficial for managing pain and function in KOA patients, these treatments are probably not suitable for those who have comorbid conditions or for long-term usage due to the minor increase in the risk of side effects, potentially outweighing their clinical benefits (da Costa et al., 2021).

Low level laser therapy (LLLT) is the electronic level absorption of laser light without the production of heat in the visible to near infrared spectral spectrum (390–1,100 nm) (Mussttaf, Jenkins, and Jha, 2019). LLLT–as a non-invasive treatment–can used for a variety of medical conditions, such as pain relief, wound healing, and mainly inflammation reduction in KOA (Tomazoni et al., 2016; Rayegani et al., 2017; Tomazoni et al., 2017). LLLT exhibited many advantages as a therapy. In conjunction with NSAIDs, LLLT reduces levels of inflammation and metalloproteinase (MP-3 and MP-13) in rats with KOA (Tomazoni et al., 2017). In addition, LLLT significantly effect in lowering the levels of pro-inflammatory cytokine expression (IL-1, IL-6, and TNF-α), myeloperoxidase, and prostaglandin E2 than NSAIDs (Tomazoni et al., 2016; Tomazoni et al., 2017). A study has applied KOA three times per week for 8 weeks to rabbits which showed that LLLT has significantly reduced MP-1 and MP-13 and slowed the loss of collagen II, aggrecan, and transforming growth factor beta; suggesting that the effects of LLLT increase over time (Wang et al., 2014).

Interestingly, major osteoarthritis treatment recommendations do not suggest LLLT. The guidelines of Osteoarthritis Research Society International (2018) underlined that LLLT should not stand alone and be regarded as a key intervention in the management of KOA (Collins, Hart, and Mills, 2019), whereas the European League Against Rheumatism osteoarthritis guidelines did not recommend LLLT as therapy for KOA (Geenen et al., 2018). This can be caused by opposition found in some systematic reviews and conflicting meta-analyses. The earliest study concluded that LLLT didn’t show expected changes to KOA patients in terms of pain intensity, severity of KOA, delayed response, stiffness, and functional outcomes (Huang et al., 2015). Next systematic review and meta-analysis found significant differences in total pain and WOMAC function, stiffness, and total on patients with LLLT compared to placebo (Rayegani et al., 2017). Last, a study in 2019 stated that LLLT significantly decreased pain and disability (Stausholm et al., 2019).

Therefore, this critical opinion aims to interpret the latest findings about the potential LLLT application in KOA and also highlight their implications and strategies in future usage.

## 2 Laser in biomedical application

Laser therapy is a non-invasive technique that promotes faster healing and tissue restoration while also helping to reduce inflammation and relieve pain because of the photobiomodulatory (PBM) effect that laser irradiation can have on cells and tissues (Dompe et al., 2020). There are various kinds of lasers available, and their applications depend on several factors, including wavelength, energy density, power output, and radiation duration. Cell growth is affected by the photobiomodulatory effect, which is mostly brought on by diode lasers with wavelengths in the red and near-infrared range (630–940 nm) while deeper penetration is achieved by using lasers with wavelengths in the “optical window” (600–1100 nm), which also causes a larger cell-light response (AlGhamdi K.M. et al., 2012). These qualities have been used to treat a variety of illnesses and ailments, including diabetes (Everett, L.A. and Paulus, Y.M., 2021), brain damage (Salehpour, F. et al., 2018), spinal cord damage (Vafei-Nezhad, S. et al., 2020), dermatological issues (Lai, D. et al., 2022), and many dental specialties (Nadhreen, A. et al., 2019). Despite many types of laser therapy, LLLT was preferred for its beneficial and safety aspects.

**FIGURE 1:**
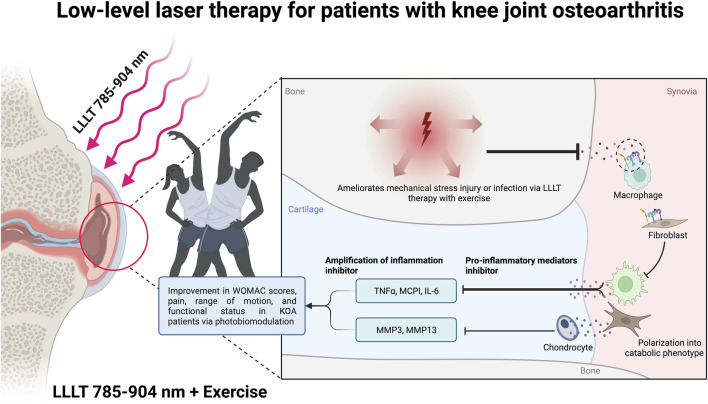
Photobiomodulation mechanism in patients with knee joint osteoarthritis by Low-level laser therapy. Created with BioRender.com premium license by Fahrul Nurkolis.

LLLT uses laser light with low energy or intensity which delivers a very small amount of energy, just enough to stimulate the target system without damaging it (Mussttaff, R.A. et al., 2019). LLLT, commonly referred to as photobiomodulation (PBM), has the ability to promote cell proliferation and improve stem cell differentiation (Figure 1) (Dompe et al., 2020). The absorption of red/near-infrared light energy, a process termed “PBM”, enhances mitochondrial ATP production, cell signaling, and growth factor synthesis, and attenuates oxidative stress (Figure 1) (Glass, G.E., 2021). Moreover, the laser makes the cell membrane hyperpolarized and activates the resynthesis of adenosine triphosphate (ATP), which gives regeneration bioprocesses free energy through hydrolysis (Ivandic, T., 2021). Due to its non-invasive nature and low risk of adverse effects, LLLT offers a distinctive strategy. It is also quite accessible, affordable, and even has the potential to be patient-directed at home (Wickenheisser, V.A. et al., 2019).

Seeing the practicality of LLLT, the LLLT technology has been commercialized and applied widely (Glass, G.E., 2021). However, the method of application of lasers varies, as well as the lack of evidence of laser type, dose distribution studies, and wavelength selection and without further rigorous and standardized research, it would create obstacles to the implementation of the LLLT (Wickenheisser, V.A. et al., 2019; Stausholm, M.B. et al., 2019). This concern remains urgent, highlighting the need for various additional preclinical and clinical studies.

## 3 Is low-level laser therapy effective on patients with knee joint osteoarthritis?

As previously noted above, and following recent evidence-based findings about the use of LLLT technology, the authors argue that LLLT technology is a promising therapeutic modality for KOA. These are the latest publications or findings regarding the use of LLLT technology which are used as the basis for the opinions stated previously.

### 3.1 *In vivo* or preclinical trials study

Prior to clinical trials, preclinical *in vivo* trials of LLLT for OA treatment have been carried out over the last few decades even since the 2000s. Study in 2016 by Mirales, L. P. et al. (2016) on KOA mouse models showed LLLT therapy at a wavelength of 808 nm successfully modulated morphological changes associated with KOA development and exerted anti-inflammatory effects on the knee of KOA mice. The 808 nm LLLT intervention was found to be more effective in repairing cartilage injury in OA experimental model, which leads to angiogenesis stimulation with inflammatory exudate reduction effect (daRosa, A.S. et al., 2012). Subsequent findings showed that anabolic and catabolic regulation may contribute to the time and site dependent positive effects of LLLT in progressive KOA. However, they stated that clinical trials were needed to confirm their study findings in human patients with progressive KOA (Wang, p. et al., 2014). A study with seventeen dogs diagnosed with KOA along with associated pain showed promising findings and suggests that LLLT may help reduce analgesic administration and improve quality of life in dogs with KOA (Barale L. et al., 2020). Lastly, to close this section, a systematic review and meta-analysis have showed reduction of several LLLT associated inflammatory biomarkers such as IL-1β, MMP-13, and TNF-α as well as their ability to modulated inflammatory cells proliferation, which further supports the use of LLLT technology as a suitable KOA therapeutic modality (Nambi G., 2021).

### 3.2 Is there sufficient clinical evidence or clinical trials?

Clinical evidence is certainly the benchmark for the feasibility of a medical intervention to be applied in the community, as well as for further developments with the goal to provide better function and efficacy. In the case of LLLT, *in vivo* studies which have been previously discussed are then further continued by researchers from various parts of the worlds in the form of clinical trials on humans. The authors have summarized the recent findings from randomized clinical trials assessing the use of LLLT therapy in KOA patients with a total sample of five hundred and two (*n* = 502), as shown in Table 1.

**TABLE 1:** Published clinical studies of low-level laser therapy on patients with knee joint osteoarthritis.

No	Studies intervention	Outcomes	References
1	LLLT Therapy on KOA (A Randomized Placebo-Controlled Trial; *n* = 50)	LLLT 904 nm + Strength Training had a positive effect on analgesic use reduction and sit-to-stand test performance improvement	Stausholm et al. (2022)
2	LLLT + exercise in KOA (A randomized controlled double-blind study; *n* = 43)	LLLT 904 nm first 3 weeks + exercise in the last 8 weeks reduced pain, disability, and drug use for 6 months period	Alfredo et al., (2022)
3	LLLT and static stretching exercises on KOA subjects (A randomised controlled trial; *n* = 215)	LLLT 904 nm + stretching exercises alleviated pain at rest, activities of daily living, stiffness, muscle shortening, and improved range of motion in KOA patients	Robbins et al. (2022)
4	LLLT on Knee Pain and Functional Status among Patients with KOA (A randomized controlled trial; *n* = 34)	LLLT 850 nm significantly improved range of motion, pain, and functional status on KOA patients	Ashraf et al., (2022)
5	LLLT and exercises in KOA Subjects (Randomized, controlled, double-blind study; *n* = 40)	LLLT 904 nm + 8-week supervised strengthening exercise program significantly reduced daily analgesic use (paracetamol)	Alfredo et al. (2018)
6	Low-level laser therapy and physical exercise pada pasien subjects with bilateral knee osteoarthritis (a blind randomized clinical trial; *n* = 120)	LLLT 808 nm + exercise showed improvements in WOMAC score and gave the best results for other gait variables (rhythm and duration of right leg support and duration of right single leg support)	de Matos Brunelli Braghin et al. (2019)
7	LLLT on pain and disability in KOA (Systematic review and meta-analysis of randomised placebo-controlled trials; *n* = 1,063)	LLLT 785–860 nm (4–8 J) and 904 nm (1–3 J) per treatment spot significantly reduced pain and disability in KOA patients	Stausholm et al. (2019)

KOA: knee osteoarthritis; LLLT: low-level laser (light) therapy; WOMAC: Western Ontario and McMaster Universities Arthritis Index.

Six recent randomized clinical trials, which have been summarized in Table 1, show that LLLT intervention has proven to be a reliable non-pharmaceutical and non-surgical treatment for KOA patients. However, most of these studies combined LLLT use with exercise. Through these findings, the authors argue that there is adequate evidence for LLLT to be further utilized, improved, and commercialized as therapy for KOA patients. LLLT therapy at wavelengths ranging 785–904 nm has been shown to improve WOMAC scores, pain, range of motion, and functional status of KOA patients. For more robust evidence, a systematic review and meta-analysis of randomized placebo-controlled trials with a total sample of 1063 showed LLLT intervention at 785–860 nm (4–8 J) and 904 nm (1–3 J) wavelengths per treatment spot significantly reduced pain and disability in KOA patients (Table 1). However, it seems that the systematic review by Huang et al. (2015) has been criticized by Stausholm et al., 2017, with a critical appraisal showing that the systematic review study featured some severe methodological deficiencies and that their meta-analysis was subject to type-II errors. Although the authors of the review study themselves have replied (Huang and Kraus, 2017), and critically refuted the critical appraisal of Stausholm et al., 2017, this later resulted in controversy in the discussion of that article or publication. Therefore, the latest systematic reviews have sprung up.

Ultimately, in line with the statement that there is a need for specific guidelines for the use of LLLT based on evidence, The American Physical Therapy Association (APTA) has the plan to communicate “Choosing Wisely” recommendations to other healthcare professionals, physical therapists, patients, and health policy stakeholders, in order to foster a conversation about the best care for specific conditions (White et al., 2015). Therefore, we believe that a guideline checklist is needed which can be used as a reference. For example, using the physician’s assessment as a basis of LLLT usage guidelines to minimize the possibility of unwanted events or side effects.

## 4 Conclusion with future implications and strategies

At the length of 785–904 nm, LLLT can be used as non-pharmaceutical and non-surgical treatment modality for KOA patients by combining it with exercises that result in improvement in WOMAC scores, pain, range of motion, and functional status in KOA patients *via* photobiomodulation (Figure 1). Through this critical opinion, the authors argue that there is sufficient evidence of LLLT to be further used and commercialized as a therapeutic option for KOA patients. However, there are variability in laser application research methodology and the lack of laser type data, dose range research and wavelength selection, and without further rigorous and standardized research, strong data may create barriers to the implementation of LLLT. Furthermore, by applying the latest technology, the authors hope and encourage that other researchers continue to develop LLLT technology in a portable manner that is easy and practical to use by the community, with protocols to regulate the use of tool based on sufficient clinical evidence.

## References

[B1] Alfredo P. P. Bjordal J. M. Junior W. S. Lopes-Martins R. Á. B. Stausholm M. B. Casarotto R. A. (2018). Long-term results of a randomized, controlled, double-blind study of low-level laser therapy before exercises in knee osteoarthritis: Laser and exercises in knee osteoarthritis. Clin. Rehabil. 32 (2), 173–178. 10.1177/0269215517723162 28776408

[B2] Alfredo P. P. Bjordal J. M. Lopes-Martins R. Á. B. Johnson M. I. Junior W. S. Marques A. P. (2022). Efficacy of prolonged application of low-level laser therapy combined with exercise in knee osteoarthritis: A randomized controlled double-blind study. Clin. Rehabil. 36 (10), 1281–1291. 10.1177/02692155221111922 35918813

[B3] AlGhamdi K. M. Kumar A. Moussa N. A. (2012). Low-level laser therapy: A useful technique for enhancing the proliferation of various cultured cells. Lasers Med. Sci. 27 (1), 237–249. 10.1007/s10103-011-0885-2 21274733

[B4] Ashraf A. Riaz S. Arslan H. M. Khan R. R. Naeem R. Malik A. (2022). Effects of low level laser therapy on knee pain and functional status among patients with knee osteoarthritis. Pak. J. Med. Health Sci. 16 (03), 863–866. 10.53350/pjmhs22163863

[B5] Barale L. Monticelli P. Raviola M. Adami C. (2020). Preliminary clinical experience of low-level laser therapy for the treatment of canine osteoarthritis-associated pain: A retrospective investigation on 17 dogs. Open Vet. J. 10 (1), 116–119. 10.4314/ovj.v10i1.16 32426264 PMC7193873

[B6] Collins N. J. Hart H. F. Mills K. A. (2019). Osteoarthritis year in review 2018: Rehabilitation and outcomes. Osteoarthr. Cartil. 27 (3), 378–391. 10.1016/j.joca.2018.11.010 30529739

[B7] Culvenor A. G. Øiestad B. E. Hart H. F. Stefanik J. J. Guermazi A. Crossley K. M. (2019). Prevalence of knee osteoarthritis features on magnetic resonance imaging in asymptomatic uninjured adults: A systematic review and meta-analysis. Br. J. Sports Med. 53 (20), 1268–1278. 10.1136/bjsports-2018-099257 29886437 PMC6837253

[B8] da Costa B. R. Pereira T. V. Saadat P. Rudnicki M. Iskander S. M. Bodmer N. S. (2021). Effectiveness and safety of non-steroidal anti-inflammatory drugs and opioid treatment for knee and hip osteoarthritis: Network meta-analysis. bmj 375, n2321. 10.1136/bmj.n2321 34642179 PMC8506236

[B9] da Rosa A. S. dos Santos A. F. da Silva M. M. Facco G. G. Perreira D. M. Alves A. C. A. (2012). Effects of low‐level laser therapy at wavelengths of 660 and 808 nm in experimental model of osteoarthritis. Photochem. Photobiol. 88 (1), 161–166. 10.1111/j.1751-1097.2011.01032.x 22053992

[B10] Dainese P. Wyngaert K. V. De Mits S. Wittoek R. Van Ginckel A. Calders P. (2021). Association between knee inflammation and knee pain in patients with knee osteoarthritis: A systematic review. Osteoarthr. Cartil., 30, (4), 516-534. 10.1016/j.joca.2021.12.003 34968719

[B11] de Matos Brunelli Braghin R. Libardi E. C. Junqueira C. Rodrigues N. C. Nogueira-Barbosa M. H. Renno A. C. M. (2019). The effect of low-level laser therapy and physical exercise on pain, stiffness, function, and spatiotemporal gait variables in subjects with bilateral knee osteoarthritis: A blind randomized clinical trial. Disabil. rehabilitation 41 (26), 3165–3172. 10.1080/09638288.2018.1493160 30324827

[B12] Dompe C. Moncrieff L. Matys J. Grzech-Leśniak K. Kocherova I. Bryja A. (2020). Photobiomodulation—Underlying mechanism and clinical applications. J. Clin. Med. 9 (6), 1724. 10.3390/jcm9061724 32503238 PMC7356229

[B13] Everett L. A. Paulus Y. M. (2021). Laser therapy in the treatment of diabetic retinopathy and diabetic macular edema. Curr. Diab. Rep. 21 (9), 35–12. 10.1007/s11892-021-01403-6 34487257 PMC8420141

[B14] Geenen R. Overman C. L. Christensen R. Åsenlöf P. Capela S. Huisinga K. L. (2018). EULAR recommendations for the health professional’s approach to pain management in inflammatory arthritis and osteoarthritis. Ann. Rheum. Dis. 77 (6), 797–807. 10.1136/annrheumdis-2017-212662 29724726

[B15] Glass G. E. (2021). Photobiomodulation: The clinical applications of low-level light therapy. Aesthet. Surg. J. 41 (6), 723–738. 10.1093/asj/sjab025 33471046

[B16] Goh S. L. Persson M. S. Stocks J. Hou Y. Lin J. Hall M. C. (2019). Efficacy and potential determinants of exercise therapy in knee and hip osteoarthritis: A systematic review and meta-analysis. Ann. Phys. rehabilitation Med. 62 (5), 356–365. 10.1016/j.rehab.2019.04.006 PMC6880792 31121333

[B17] Huang Z. Chen J. Ma J. Shen B. Pei F. Kraus V. B. (2015). Effectiveness of low-level laser therapy in patients with knee osteoarthritis: A systematic review and meta-analysis. Osteoarthr. Cartil. 23 (9), 1437–1444. 10.1016/j.joca.2015.04.005 PMC4814167 25914044

[B18] Huang Z. Y. Kraus V. B. (2017). Reply to Stausholm et al.'s letter to the editor regarding our published study entitled, "effectiveness of low-level laser therapy in patients with knee osteoarthritis: A systematic review and meta-analysis. Osteoarthr. Cartil. 25 (4), e11–e14. 10.1016/j.joca.2016.10.015 27816574

[B19] Ivandic T. (2021). Low-level laser therapy. Dtsch. Arztebl. Int. 118 (5), 69. 10.3238/arztebl.m2021.0034 PMC8188418 33785126

[B20] Jang S. Lee K. Ju J. H. (2021). Recent updates of diagnosis, pathophysiology, and treatment on osteoarthritis of the knee. Int. J. Mol. Sci. 22 (5), 2619. 10.3390/ijms22052619 33807695 PMC7961389

[B21] Lai D. Zhou S. Cheng S. Liu H. Cui Y. (2022). Laser therapy in the treatment of melasma: A systematic review and meta-analysis. Lasers Med. Sci. 37, 2099–2110. 10.1007/s10103-022-03514-2 35122202

[B22] Milares L. P. Assis L. Siqueira A. Claudino V. Domingos H. Almeida T. (2016). Effectiveness of an aquatic exercise program and low-level laser therapy on articular cartilage in an experimental model of osteoarthritis in rats. Connect. Tissue Res. 57 (5), 398–407. 10.1080/03008207.2016.1193174 27220395

[B23] Mora J. C. Przkora R. Cruz-Almeida Y. (2018). Knee osteoarthritis: Pathophysiology and current treatment modalities. J. Pain Res. 11, 2189–2196. 10.2147/JPR.S154002 30323653 PMC6179584

[B24] Mussttaf R. A. Jenkins D. F. Jha A. N. (2019). Assessing the impact of low level laser therapy (LLLT) on biological systems: A review. Int. J. Radiat. Biol. 95 (2), 120–143. 10.1080/09553002.2019.1524944 30614743

[B25] Nadhreen A. Alamoudi N. Elkhodary H. (2019). Low-level laser therapy in dentistry: Extra-oral applications. Niger. J. Clin. Pract. 22 (10), 1313. 10.4103/njcp.njcp_53_19 31607717

[B26] Nambi G. (2021). Does low level laser therapy has effects on inflammatory biomarkers IL-1β, IL-6, TNF-α, and MMP-13 in osteoarthritis of rat models—A systemic review and meta-analysis. Lasers Med. Sci. 36 (3), 475–484. 10.1007/s10103-020-03124-w 32833088

[B27] Rannou F. Pelletier J. P. Martel-Pelletier J. (2016)., Efficacy and safety of topical NSAIDs in the management of osteoarthritis: Evidence from real-life setting trials and surveys, Seminars arthritis rheumatism, 45. WB Saunders, S18–S21. 10.1016/j.semarthrit.2015.11.007 4 26806189

[B28] Rayegani S. M. Raeissadat S. A. Heidari S. Moradi-Joo M. (2017). Safety and effectiveness of low-level laser therapy in patients with knee osteoarthritis: A systematic review and meta-analysis. J. Lasers Med. Sci. 8 (1), S12–S19. 10.15171/jlms.2017.s3 29071029 PMC5642172

[B29] Robbins S. R. Alfredo P. P. Junior W. S. Marques A. P. (2022). Low-level laser therapy and static stretching exercises for patients with knee osteoarthritis: A randomised controlled trial. Clin. Rehabil. 36 (2), 204–213. 10.1177/02692155211047017 34714175

[B30] Salehpour F. Mahmoudi J. Kamari F. Sadigh-Eteghad S. Rasta S. H. Hamblin M. R. (2018). Brain photobiomodulation therapy: A narrative review. Mol. Neurobiol. 55 (8), 6601–6636. 10.1007/s12035-017-0852-4 29327206 PMC6041198

[B31] Stausholm M. B. Bjordal J. M. Lopes-Martins R. A. B. Joensen J. (2017). Methodological flaws in meta-analysis of low-level laser therapy in knee osteoarthritis: A letter to the editor. Osteoarthritis and cartilage. Apr 25 (4), e9–e10. 10.1016/j.joca.2016.09.022 27816573

[B32] Stausholm M. B. Naterstad I. F. Alfredo P. P. Couppé C. Fersum K. V. Leal-Junior E. C. P. (2022). Short-and long-term effectiveness of low-level laser therapy combined with strength training in knee osteoarthritis: A randomized placebo-controlled trial. J. Clin. Med. 11 (12), 3446. 10.3390/jcm11123446 35743513 PMC9225274

[B33] Stausholm M. B. Naterstad I. F. Joensen J. Lopes-Martins R. Á. B. Sæbø H. Lund H. (2019). Efficacy of low-level laser therapy on pain and disability in knee osteoarthritis: Systematic review and meta-analysis of randomised placebo-controlled trials. BMJ open 9 (10), e031142. 10.1136/bmjopen-2019-031142 PMC6830679 31662383

[B34] Tomazoni S. S. Leal-Junior E. C. P. Frigo L. Pallotta R. C. Teixeira S. De Almeida P. (2016). Isolated and combined effects of photobiomodulation therapy, topical nonsteroidal anti-inflammatory drugs, and physical activity in the treatment of osteoarthritis induced by papain. J. Biomed. Opt. 21 (10), 108001. 10.1117/1.JBO.21.10.108001 27752702

[B35] Tomazoni S. S. Leal-Junior E. C. P. Pallotta R. C. Teixeira S. de Almeida P. Lopes-Martins R. Á. B. (2017). Effects of photobiomodulation therapy, pharmacological therapy, and physical exercise as single and/or combined treatment on the inflammatory response induced by experimental osteoarthritis. Lasers Med. Sci. 32 (1), 101–108. 10.1007/s10103-016-2091-8 27726040

[B36] Vafaei-Nezhad S. Hassan M. P. Noroozian M. Aliaghaei A. Tehrani A. S. Abbaszadeh H. A. (2020). A review of low-level laser therapy for spinal cord injury: Challenges and safety. J. Lasers Med. Sci. 11 (4), 363–368. 10.34172/jlms.2020.59 33425285 PMC7736940

[B37] Vitaloni M. Botto-van Bemden A. Sciortino Contreras R. M. Scotton D. Bibas M. Quintero M. (2019). Global management of patients with knee osteoarthritis begins with quality of life assessment: A systematic review. BMC Musculoskelet. Disord. 20 (1), 493–504. 10.1186/s12891-019-2895-3 31656197 PMC6815415

[B38] Vos T. Allen C. Arora M. Barber R. M. Bhutta Z. A. Brown A. (2016). Global, regional, and national incidence, prevalence, and years lived with disability for 310 diseases and injuries, 1990–2015: A systematic analysis for the global burden of disease study 2015. lancet 388 (10053), 1545–1602. 10.1016/S0140-6736(16)31678-6 27733282 PMC5055577

[B39] Wang P. Liu C. Yang X. Zhou Y. Wei X. Ji Q. (2014). Effects of low-level laser therapy on joint pain, synovitis, anabolic, and catabolic factors in a progressive osteoarthritis rabbit model. Lasers Med. Sci. 29 (6), 1875–1885. 10.1007/s10103-014-1600-x 24890034

[B40] White N. T. Delitto A. Manal T. J. Miller S. (2015). The American Physical Therapy Association's top five choosing wisely recommendations. Phys. Ther. 95 (1), 9–24. 10.2522/ptj.20140287 25223237

[B41] Wickenheisser V. A. Zywot E. M. Rabjohns E. M. Lee H. H. Lawrence D. S. Tarrant T. K. (2019). Laser light therapy in inflammatory, musculoskeletal, and autoimmune disease. Curr. Allergy Asthma Rep. 19 (8), 37–15. 10.1007/s11882-019-0869-z 31267251 PMC7357616

